# *N*,*N*,*N*′,*N*′,*N*′′-Penta­methyl-*N*′′-[3-(tri­methyl­aza­nium­yl)prop­yl]guanidinium bis­(tetra­phenyl­borate)

**DOI:** 10.1107/S1600536813001992

**Published:** 2013-01-26

**Authors:** Ioannis Tiritiris

**Affiliations:** a Fakultät Chemie/Organische Chemie, Hochschule Aalen, Beethovenstrasse 1, D-73430 Aalen, Germany

## Abstract

In the crystal structure of the title salt, C_12_H_30_N_4_^2+^·2C_24_H_20_B^−^, the C—N bond lengths in the central CN_3_ unit of the guanidinium ion are 1.3388 (17), 1.3390 (16) and 1.3540 (17) Å, indicating partial double-bond character in each. The central C atom is bonded to the three N atoms in a nearly ideal trigonal-planar geometry and the positive charge is delocalized in the CN_3_ plane. The bonds between the N atoms and the terminal *C*-methyl groups of the guanidinium moiety, all have values close to a typical single bond [1.4630 (16)–1.4697 (17) Å]. C—H⋯π inter­actions are present between the guanidinium H atoms and the phenyl C atoms of one tetra­phenyl­borate ion. The phenyl rings form a kind of aromatic pocket, in which the guanidinium ion is embedded.

## Related literature

For the synthesis of *N′′*-[3-(dimethyl­amino)­prop­yl]- *N*,*N*,*N′*,*N′*-tetra­methyl­guanidine, see: Tiritiris & Kantlehner (2012 ▶). For the crystal structures of alkali metal tetra­phenyl­borates, see: Behrens *et al.* (2012 ▶).
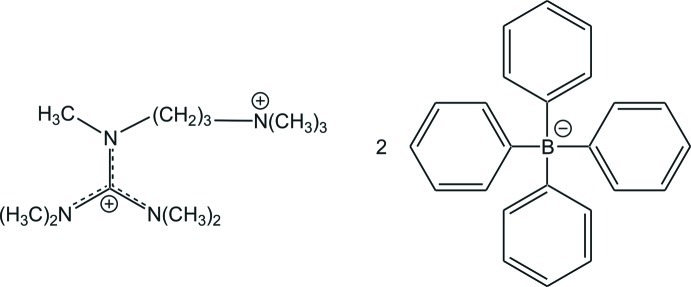


## Experimental

### 

#### Crystal data

C_12_H_30_N_4_^+^·2C_24_H_20_B^−^
*M* *_r_* = 868.82
Monoclinic, 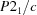

*a* = 17.7622 (4) Å
*b* = 16.1667 (3) Å
*c* = 17.3787 (4) Å
β = 98.045 (1)°
*V* = 4941.29 (18) Å^3^
*Z* = 4
Mo *K*α radiation
μ = 0.07 mm^−1^
*T* = 100 K
0.27 × 0.25 × 0.20 mm

#### Data collection

Bruker–Nonius KappaCCD diffractometer
22713 measured reflections
12056 independent reflections
8821 reflections with *I* > 2σ(*I*)
*R*_int_ = 0.038

#### Refinement

*R*[*F* ^2^ > 2σ(*F* ^2^)] = 0.045
*wR*(*F* ^2^) = 0.108
*S* = 1.02
12056 reflections
603 parameters
H-atom parameters constrained
Δρ_max_ = 0.31 e Å^−3^
Δρ_min_ = −0.27 e Å^−3^

### 

Data collection: *COLLECT* (Hooft, 2004 ▶); cell refinement: *SCALEPACK* (Otwinowski & Minor, 1997 ▶); data reduction: *SCALEPACK*; program(s) used to solve structure: *SHELXS97* (Sheldrick, 2008 ▶); program(s) used to refine structure: *SHELXL97* (Sheldrick, 2008 ▶); molecular graphics: *DIAMOND* (Brandenburg & Putz, 2005 ▶); software used to prepare material for publication: *SHELXL97*.

## Supplementary Material

Crystal structure: contains datablock(s) I, global. DOI: 10.1107/S1600536813001992/zl2530sup1.cif

Structure factors: contains datablock(s) I. DOI: 10.1107/S1600536813001992/zl2530Isup2.hkl

Additional supplementary materials:  crystallographic information; 3D view; checkCIF report

## Figures and Tables

**Table 1:** Hydrogen-bond geometry (Å, °) *Cg*1, *Cg*4, *Cg*5, *Cg*6, and *Cg*8 are the centroids of the C13–C18,C31–C36, C37–C42, C43–C48 and C55–C60 rings, respectively.

*D*—H⋯*A*	*D*—H	H⋯*A*	*D*⋯*A*	*D*—H⋯*A*
C3—H3*C*⋯*Cg*7	0.98	2.62	3.3783 (16)	134
C7—H7*B*⋯*Cg*1	0.99	2.80	3.7805 (14)	169
C9—H9*B*⋯*Cg*6	0.99	2.52	3.4075 (14)	149
C11—H11*C*⋯*Cg*5^i^	0.98	2.62	3.4852 (15)	147
C12—H12*A*⋯*Cg*4	0.98	2.59	3.4044 (15)	141
C12—H12*B*⋯*Cg*8^ii^	0.98	2.69	3.5990 (15)	155

Symmetry codes: (i) 
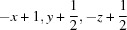
; (ii) 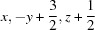.

**Fig. 1.**
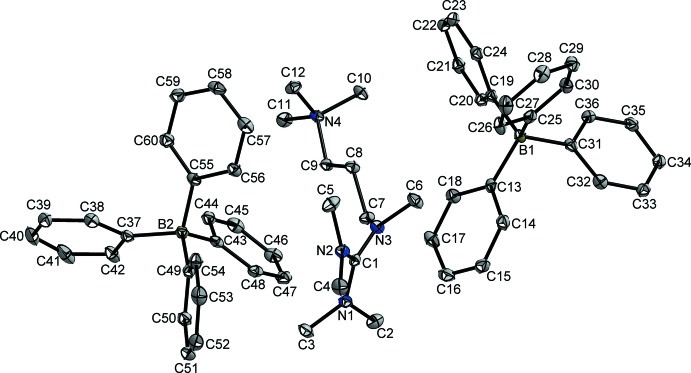
The structure of the title compound with displacement ellipsoids at the 50% probability level. All hydrogen atoms were omitted for the sake of clarity.

**Fig. 2.**
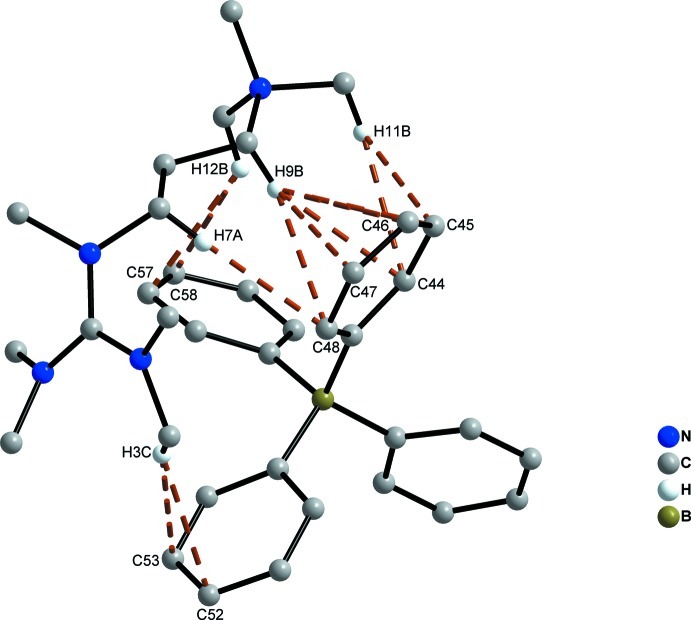
C–H···π interactions (brown dashed lines) between the hydrogen atoms of the guanidinium ion and the phenyl carbon atoms of one tetraphenylborate ion.

## supplementary crystallographic information

### Comment

Molecules in which alkylamino groups are connected with a guanidine function represent promising candidates for CO_2_ capture, since in such type of compounds two nitrogen centers with different basicity are present, which can react with CO_2_. Guanidines with additional basic nitrogen functions like tertiary amino groups are well known in the literature (Tiritiris & Kantlehner, 2012), except of their peralkylated guanidinium salts. By alkylation of the corresponding aminoguanidine with dimethyl sulfate and subsequent anion exchange, it was possible to obtain the here presented title compound. According to the structure analysis, the C1–N1 bond of the the CN_3_ unit is 1.3390 (16) Å, C1–N2 = 1.3388 (17) Å and C1–N3 = 1.3540 (17) Å, showing partial double-bond character. The N–C1–N angles are: 121.12 (12)° (N1–C1–N2), 120.28 (12)° (N1–C1–N3) and 118.60 (11)° (N2–C1–N3), which indicates a nearly ideal trigonal-planar surrounding of the carbon centre by the nitrogen atoms. The positive charge is completely delocalized on the CN_3_ plane (Fig. 1). The bonds between the N atoms and the terminal *C*-methyl groups of the guanidinium moiety all have values close to a typical single bond [1.4630 (16)–1.4697 (17) Å]. The C–N bond lengths in the terminal trimethylammonium group are slightly elongated [1.4983 (17)–1.5171 (16) Å]. The bond lengths and angles in the tetraphenylborate ions are in good agreement with the data from the crystal structure analysis of the alkali metal tetraphenylborates (Behrens *et al.*, 2012). C–H···π interactions between the hydrogen atoms of –N(CH_3_)_2_, –CH_2_ and –N^+^(CH_3_)_3_ groups of the guanidinium ion and the phenyl carbon atoms of only one tetraphenylborate ion are mainly present, ranging from 2.724 (2) to 2.895 (2) Å (Fig. 2).

### Experimental

The title compound was obtained by reaction of *N*''-[3-(dimethylamino)propyl]-*N*,*N*,*N*',*N*'-tetramethylguanidine (Tiritiris & Kantlehner, 2012) with two equivalents dimethyl sulfate in acetonitrile at room temperature. After evaporation of the solvent the crude *N*,*N*,*N*',*N*',*N*''-pentamethyl-*N*''-[3-(trimethylazaniumyl)propyl]-guanidinium bis(methylsulfate) (I) was washed with diethylether and dried *in vacuo*. 1.0 g (2.2 mmol) of (I) was dissolved in 20 ml acetonitrile and 1.51 g (4.4 mmol) of sodium tetraphenylborate in 20 ml acetonitrile were added. After stirring for one hour at room temperature, the precipitated sodium methylsulfate was filtered off. The title compound crystallized from a saturated acetone solution after several days at 273 K, forming colorless single crystals. Yield: 1.34 g (68.2%). ^1^H NMR (500 MHz, CD_3_CN/TMS): δ = 2.10 (broad s, 1 H, –CH_2_), 2.35 (broad s, 1 H, –CH_2_), 2.95 (s, 3 H, –NCH_3_), 2.98 [s, 12 H, –N(CH_3_)_2_], 3.13 [s, 9 H, –N^+^(CH_3_)_3_], 3.20–3.40 (m, 4 H, –CH_2_), 6.86–6.91 (t, 8 H, –C_6_H_5_), 6.96–7.04 (t, 16 H,–C_6_H_5_), 7.25–7.30 (m, 16 H, –C_6_H_5_). ^13^C NMR (125 MHz, CD_3_CN/TMS): δ = 22.5 (–CH_2_), 37.9 (–NCH_3_), 40.5 [–N(CH_3_)_2_], 49.8 (–CH_2_), 53.6–53.9 [–N^+^(CH_3_)_3_], 64.3 (–CH_2_), 122.3 (–C_6_H_5_), 126.1 – 126.7 (–C_6_H_5_), 137.0 (–C_6_H_5_), 162.9 – 164.0 (–C_6_H_5_), 165.5 (N_3_C^+^).

### Refinement

The hydrogen atoms of the methyl groups were allowed to rotate with a fixed angle around the C–N bond to best fit the experimental electron density, with *U*(H) set to 1.5 *U*_eq_(C) and d(C—H) = 0.98 Å. The remaining H atoms were placed in calculated positions with d(C—H) = 0.99 Å (H atoms in CH_2_ groups) and (C—H) = 0.95 Å (H atoms in aromatic rings). They were included in the refinement in the riding model approximation, with *U*(H) set to 1.2 *U*_eq_(C).

### Crystal data

**Table:**

C_12_H_30_N_4_^+^·2C_24_H_20_B^−^	*F*(000) = 1872
*M**_r_* = 868.82	*D*_x_ = 1.168 Mg m^−^^3^
Monoclinic, *P*2_1_/*c*	Melting point: 502 K
Hall symbol: -P 2ybc	Mo *K*α radiation, λ = 0.71073 Å
*a* = 17.7622 (4) Å	Cell parameters from 11929 reflections
*b* = 16.1667 (3) Å	θ = 0.4–28.3°
*c* = 17.3787 (4) Å	µ = 0.07 mm^−^^1^
β = 98.045 (1)°	*T* = 100 K
*V* = 4941.29 (18) Å^3^	Polyhedral, colorless
*Z* = 4	0.27 × 0.25 × 0.20 mm

### Data collection

**Table:**

Bruker–Nonius KappaCCD diffractometer	8821 reflections with *I* > 2σ(*I*)
Radiation source: sealed tube	*R*_int_ = 0.038
Graphite monochromator	θ_max_ = 28.3°, θ_min_ = 1.2°
φ scans, and ω scans	*h* = −23→23
22713 measured reflections	*k* = −21→21
12056 independent reflections	*l* = −23→23

### Refinement

**Table:**

Refinement on *F*^2^	Primary atom site location: structure-invariant direct methods
Least-squares matrix: full	Secondary atom site location: difference Fourier map
*R*[*F*^2^ > 2σ(*F*^2^)] = 0.045	Hydrogen site location: difference Fourier map
*wR*(*F*^2^) = 0.108	H-atom parameters constrained
*S* = 1.02	*w* = 1/[σ^2^(*F*_o_^2^) + (0.0389*P*)^2^ + 2.0808*P*] where *P* = (*F*_o_^2^ + 2*F*_c_^2^)/3
12056 reflections	(Δ/σ)_max_ < 0.001
603 parameters	Δρ_max_ = 0.31 e Å^−^^3^
0 restraints	Δρ_min_ = −0.27 e Å^−^^3^

### Special details

**Table:**

Geometry. All e.s.d.'s (except the e.s.d. in the dihedral angle between two l.s. planes) are estimated using the full covariance matrix. The cell e.s.d.'s are taken into account individually in the estimation of e.s.d.'s in distances, angles and torsion angles; correlations between e.s.d.'s in cell parameters are only used when they are defined by crystal symmetry. An approximate (isotropic) treatment of cell e.s.d.'s is used for estimating e.s.d.'s involving l.s. planes.
Refinement. Refinement of *F*^2^ against ALL reflections. The weighted *R*-factor *wR* and goodness of fit *S* are based on *F*^2^, conventional *R*-factors *R* are based on *F*, with *F* set to zero for negative *F*^2^. The threshold expression of *F*^2^ > σ(*F*^2^) is used only for calculating *R*-factors(gt) *etc*. and is not relevant to the choice of reflections for refinement. *R*-factors based on *F*^2^ are statistically about twice as large as those based on *F*, and *R*- factors based on ALL data will be even larger.

### Fractional atomic coordinates and isotropic or equivalent isotropic displacement parameters (Å^2^)

**Table:**

	*x*	*y*	*z*	*U*_iso_*/*U*_eq_	
N1	0.72188 (6)	0.33127 (7)	0.03324 (6)	0.0155 (2)	
N2	0.82034 (6)	0.28130 (7)	0.12425 (6)	0.0163 (2)	
N3	0.81518 (6)	0.42077 (7)	0.09366 (6)	0.0152 (2)	
N4	0.73663 (6)	0.66166 (7)	0.24912 (6)	0.0134 (2)	
C1	0.78526 (7)	0.34387 (8)	0.08336 (7)	0.0136 (2)	
C2	0.70205 (8)	0.38292 (9)	−0.03593 (8)	0.0205 (3)	
H2A	0.6572	0.4163	−0.0300	0.031*	
H2B	0.6909	0.3475	−0.0819	0.031*	
H2C	0.7448	0.4195	−0.0422	0.031*	
C3	0.66849 (8)	0.26374 (9)	0.04174 (9)	0.0216 (3)	
H3A	0.6774	0.2183	0.0068	0.032*	
H3B	0.6162	0.2839	0.0285	0.032*	
H3C	0.6762	0.2439	0.0956	0.032*	
C4	0.82067 (8)	0.19610 (9)	0.09563 (9)	0.0211 (3)	
H4A	0.7872	0.1620	0.1229	0.032*	
H4B	0.8725	0.1741	0.1051	0.032*	
H4C	0.8025	0.1952	0.0397	0.032*	
C5	0.86344 (8)	0.29427 (10)	0.20146 (8)	0.0231 (3)	
H5A	0.9179	0.2887	0.1986	0.035*	
H5B	0.8482	0.2530	0.2376	0.035*	
H5C	0.8531	0.3498	0.2199	0.035*	
C6	0.89702 (7)	0.43361 (9)	0.09621 (9)	0.0208 (3)	
H6A	0.9211	0.3816	0.0838	0.031*	
H6B	0.9188	0.4518	0.1484	0.031*	
H6C	0.9060	0.4759	0.0582	0.031*	
C7	0.76724 (7)	0.49447 (8)	0.09672 (7)	0.0156 (3)	
H7A	0.7133	0.4773	0.0920	0.019*	
H7B	0.7731	0.5309	0.0521	0.019*	
C8	0.78810 (7)	0.54320 (8)	0.17278 (7)	0.0153 (3)	
H8A	0.7989	0.5045	0.2171	0.018*	
H8B	0.8341	0.5771	0.1700	0.018*	
C9	0.72114 (7)	0.59887 (8)	0.18394 (7)	0.0146 (2)	
H9A	0.7050	0.6289	0.1348	0.017*	
H9B	0.6781	0.5635	0.1942	0.017*	
C10	0.78839 (8)	0.72864 (8)	0.22812 (8)	0.0183 (3)	
H10A	0.7986	0.7678	0.2714	0.027*	
H10B	0.7641	0.7578	0.1817	0.027*	
H10C	0.8364	0.7042	0.2175	0.027*	
C11	0.66238 (7)	0.70012 (9)	0.26196 (8)	0.0183 (3)	
H11A	0.6719	0.7443	0.3008	0.027*	
H11B	0.6297	0.6579	0.2806	0.027*	
H11C	0.6371	0.7233	0.2129	0.027*	
C12	0.77035 (8)	0.62061 (9)	0.32345 (7)	0.0176 (3)	
H12A	0.8213	0.6001	0.3183	0.026*	
H12B	0.7380	0.5742	0.3346	0.026*	
H12C	0.7738	0.6607	0.3661	0.026*	
B1	0.92120 (8)	0.77122 (9)	0.01258 (8)	0.0130 (3)	
C13	0.84884 (7)	0.70967 (8)	−0.01978 (7)	0.0139 (2)	
C14	0.85773 (8)	0.64051 (9)	−0.06727 (8)	0.0197 (3)	
H14A	0.9076	0.6256	−0.0759	0.024*	
C15	0.79684 (9)	0.59327 (9)	−0.10199 (8)	0.0230 (3)	
H15A	0.8058	0.5482	−0.1347	0.028*	
C16	0.72308 (9)	0.61134 (9)	−0.08931 (8)	0.0222 (3)	
H16A	0.6812	0.5800	−0.1138	0.027*	
C17	0.71217 (8)	0.67606 (9)	−0.04010 (8)	0.0211 (3)	
H17A	0.6624	0.6884	−0.0292	0.025*	
C18	0.77377 (8)	0.72337 (9)	−0.00630 (8)	0.0172 (3)	
H18A	0.7644	0.7671	0.0276	0.021*	
C19	0.90010 (7)	0.83216 (8)	0.08230 (7)	0.0131 (2)	
C20	0.84233 (7)	0.89228 (8)	0.06892 (7)	0.0148 (2)	
H20A	0.8138	0.8959	0.0186	0.018*	
C21	0.82520 (7)	0.94659 (8)	0.12597 (8)	0.0170 (3)	
H21A	0.7862	0.9867	0.1139	0.020*	
C22	0.86491 (8)	0.94244 (9)	0.20072 (8)	0.0180 (3)	
H22A	0.8529	0.9788	0.2402	0.022*	
C23	0.92215 (8)	0.88448 (9)	0.21657 (8)	0.0179 (3)	
H23A	0.9499	0.8810	0.2673	0.021*	
C24	0.93943 (7)	0.83101 (8)	0.15836 (7)	0.0157 (3)	
H24A	0.9795	0.7922	0.1707	0.019*	
C25	0.99644 (7)	0.71673 (8)	0.04716 (7)	0.0144 (2)	
C26	0.99181 (8)	0.63813 (9)	0.08039 (8)	0.0189 (3)	
H26A	0.9434	0.6125	0.0778	0.023*	
C27	1.05539 (8)	0.59606 (9)	0.11711 (8)	0.0212 (3)	
H27A	1.0499	0.5426	0.1383	0.025*	
C28	1.12671 (8)	0.63228 (9)	0.12264 (8)	0.0201 (3)	
H28A	1.1702	0.6042	0.1480	0.024*	
C29	1.13376 (8)	0.71018 (9)	0.09060 (8)	0.0186 (3)	
H29A	1.1823	0.7357	0.0941	0.022*	
C30	1.06967 (7)	0.75080 (8)	0.05334 (8)	0.0163 (3)	
H30A	1.0758	0.8037	0.0312	0.020*	
C31	0.94239 (7)	0.82836 (8)	−0.06068 (7)	0.0135 (2)	
C32	0.95366 (7)	0.79298 (8)	−0.13195 (8)	0.0159 (3)	
H32A	0.9483	0.7348	−0.1381	0.019*	
C33	0.97242 (7)	0.83945 (9)	−0.19412 (7)	0.0170 (3)	
H33A	0.9794	0.8126	−0.2413	0.020*	
C34	0.98100 (7)	0.92435 (9)	−0.18763 (8)	0.0179 (3)	
H34A	0.9929	0.9564	−0.2302	0.022*	
C35	0.97190 (8)	0.96169 (9)	−0.11763 (8)	0.0189 (3)	
H35A	0.9786	1.0197	−0.1117	0.023*	
C36	0.95301 (7)	0.91444 (8)	−0.05604 (7)	0.0161 (3)	
H36A	0.9470	0.9416	−0.0088	0.019*	
B2	0.56950 (8)	0.36867 (9)	0.27014 (8)	0.0139 (3)	
C37	0.49422 (7)	0.35924 (8)	0.31507 (8)	0.0165 (3)	
C38	0.49765 (8)	0.31050 (9)	0.38226 (8)	0.0215 (3)	
H38A	0.5429	0.2803	0.3990	0.026*	
C39	0.43758 (9)	0.30464 (10)	0.42539 (9)	0.0284 (3)	
H39A	0.4426	0.2718	0.4711	0.034*	
C40	0.37027 (9)	0.34683 (10)	0.40166 (10)	0.0325 (4)	
H40A	0.3297	0.3449	0.4319	0.039*	
C41	0.36309 (9)	0.39164 (10)	0.33346 (11)	0.0300 (4)	
H41A	0.3165	0.4188	0.3154	0.036*	
C42	0.42396 (8)	0.39720 (9)	0.29082 (9)	0.0213 (3)	
H42A	0.4175	0.4278	0.2437	0.026*	
C43	0.55692 (7)	0.44382 (8)	0.20609 (7)	0.0147 (3)	
C44	0.53044 (7)	0.52126 (8)	0.22770 (8)	0.0168 (3)	
H44A	0.5221	0.5290	0.2800	0.020*	
C45	0.51585 (7)	0.58709 (9)	0.17622 (8)	0.0188 (3)	
H45A	0.4956	0.6374	0.1929	0.023*	
C46	0.53105 (8)	0.57886 (9)	0.10041 (8)	0.0194 (3)	
H46A	0.5213	0.6233	0.0646	0.023*	
C47	0.56067 (7)	0.50486 (9)	0.07758 (8)	0.0185 (3)	
H47A	0.5731	0.4993	0.0264	0.022*	
C48	0.57231 (7)	0.43850 (9)	0.12931 (7)	0.0159 (3)	
H48A	0.5913	0.3880	0.1118	0.019*	
C49	0.58100 (7)	0.27853 (8)	0.22960 (7)	0.0149 (3)	
C50	0.52557 (8)	0.25007 (9)	0.16949 (8)	0.0182 (3)	
H50A	0.4836	0.2849	0.1520	0.022*	
C51	0.52972 (8)	0.17294 (9)	0.13454 (8)	0.0216 (3)	
H51A	0.4911	0.1564	0.0941	0.026*	
C52	0.59017 (9)	0.12011 (9)	0.15866 (8)	0.0229 (3)	
H52A	0.5938	0.0680	0.1342	0.027*	
C53	0.64505 (9)	0.14484 (9)	0.21901 (9)	0.0222 (3)	
H53A	0.6860	0.1089	0.2371	0.027*	
C54	0.64020 (8)	0.22249 (9)	0.25323 (8)	0.0182 (3)	
H54A	0.6786	0.2381	0.2943	0.022*	
C55	0.64417 (7)	0.39616 (8)	0.33197 (7)	0.0149 (3)	
C56	0.71816 (7)	0.38922 (8)	0.31254 (8)	0.0166 (3)	
H56A	0.7243	0.3689	0.2625	0.020*	
C57	0.78282 (8)	0.41093 (8)	0.36350 (8)	0.0183 (3)	
H57A	0.8318	0.4035	0.3485	0.022*	
C58	0.77572 (8)	0.44333 (8)	0.43604 (8)	0.0193 (3)	
H58A	0.8196	0.4572	0.4715	0.023*	
C59	0.70356 (8)	0.45515 (9)	0.45582 (8)	0.0205 (3)	
H59A	0.6977	0.4794	0.5044	0.025*	
C60	0.63950 (8)	0.43173 (9)	0.40482 (8)	0.0186 (3)	
H60A	0.5907	0.4402	0.4200	0.022*	

### Atomic displacement parameters (Å^2^)

**Table:**

	*U*^11^	*U*^22^	*U*^33^	*U*^12^	*U*^13^	*U*^23^
N1	0.0156 (5)	0.0144 (5)	0.0166 (5)	−0.0024 (4)	0.0024 (4)	−0.0029 (4)
N2	0.0192 (6)	0.0131 (5)	0.0168 (5)	−0.0002 (4)	0.0038 (4)	−0.0011 (4)
N3	0.0128 (5)	0.0127 (5)	0.0204 (5)	0.0001 (4)	0.0036 (4)	−0.0029 (4)
N4	0.0144 (5)	0.0099 (5)	0.0154 (5)	0.0000 (4)	0.0009 (4)	−0.0007 (4)
C1	0.0141 (6)	0.0144 (6)	0.0135 (6)	−0.0014 (5)	0.0061 (5)	−0.0028 (5)
C2	0.0220 (7)	0.0228 (7)	0.0161 (6)	0.0030 (6)	0.0009 (5)	−0.0023 (5)
C3	0.0171 (7)	0.0188 (7)	0.0290 (7)	−0.0066 (5)	0.0041 (5)	−0.0073 (6)
C4	0.0253 (7)	0.0116 (6)	0.0272 (7)	0.0014 (5)	0.0072 (6)	−0.0014 (5)
C5	0.0255 (7)	0.0260 (8)	0.0170 (6)	0.0066 (6)	0.0004 (5)	−0.0008 (6)
C6	0.0135 (6)	0.0182 (7)	0.0318 (7)	−0.0025 (5)	0.0064 (5)	−0.0047 (6)
C7	0.0167 (6)	0.0138 (6)	0.0163 (6)	0.0020 (5)	0.0022 (5)	−0.0027 (5)
C8	0.0157 (6)	0.0135 (6)	0.0164 (6)	−0.0003 (5)	0.0013 (5)	−0.0022 (5)
C9	0.0156 (6)	0.0137 (6)	0.0141 (6)	−0.0016 (5)	0.0007 (5)	−0.0031 (5)
C10	0.0192 (6)	0.0125 (6)	0.0232 (7)	−0.0039 (5)	0.0029 (5)	0.0014 (5)
C11	0.0170 (6)	0.0158 (7)	0.0222 (6)	0.0030 (5)	0.0034 (5)	−0.0036 (5)
C12	0.0219 (7)	0.0166 (6)	0.0137 (6)	0.0016 (5)	0.0001 (5)	0.0013 (5)
B1	0.0133 (6)	0.0110 (7)	0.0149 (6)	−0.0004 (5)	0.0025 (5)	0.0008 (5)
C13	0.0177 (6)	0.0116 (6)	0.0125 (5)	−0.0011 (5)	0.0028 (5)	0.0028 (5)
C14	0.0229 (7)	0.0159 (7)	0.0219 (7)	−0.0038 (5)	0.0090 (5)	−0.0007 (5)
C15	0.0356 (8)	0.0172 (7)	0.0172 (6)	−0.0076 (6)	0.0078 (6)	−0.0038 (5)
C16	0.0268 (7)	0.0189 (7)	0.0188 (6)	−0.0087 (6)	−0.0045 (5)	0.0033 (5)
C17	0.0156 (6)	0.0201 (7)	0.0266 (7)	−0.0008 (5)	−0.0004 (5)	0.0035 (6)
C18	0.0191 (6)	0.0141 (6)	0.0183 (6)	0.0003 (5)	0.0023 (5)	0.0005 (5)
C19	0.0147 (6)	0.0104 (6)	0.0147 (6)	−0.0025 (5)	0.0039 (5)	0.0018 (5)
C20	0.0151 (6)	0.0140 (6)	0.0149 (6)	−0.0019 (5)	0.0008 (5)	0.0010 (5)
C21	0.0160 (6)	0.0144 (6)	0.0215 (6)	0.0000 (5)	0.0055 (5)	−0.0001 (5)
C22	0.0212 (7)	0.0174 (7)	0.0166 (6)	−0.0047 (5)	0.0074 (5)	−0.0031 (5)
C23	0.0201 (6)	0.0197 (7)	0.0137 (6)	−0.0053 (5)	0.0023 (5)	0.0018 (5)
C24	0.0171 (6)	0.0135 (6)	0.0165 (6)	−0.0019 (5)	0.0025 (5)	0.0037 (5)
C25	0.0170 (6)	0.0122 (6)	0.0147 (6)	0.0016 (5)	0.0044 (5)	−0.0003 (5)
C26	0.0182 (6)	0.0153 (7)	0.0242 (7)	−0.0001 (5)	0.0067 (5)	0.0036 (5)
C27	0.0240 (7)	0.0147 (7)	0.0259 (7)	0.0033 (5)	0.0076 (6)	0.0062 (5)
C28	0.0190 (7)	0.0210 (7)	0.0204 (6)	0.0071 (5)	0.0030 (5)	0.0026 (5)
C29	0.0149 (6)	0.0201 (7)	0.0210 (6)	0.0004 (5)	0.0030 (5)	−0.0014 (5)
C30	0.0181 (6)	0.0132 (6)	0.0182 (6)	0.0007 (5)	0.0047 (5)	0.0011 (5)
C31	0.0105 (6)	0.0146 (6)	0.0150 (6)	−0.0004 (5)	0.0001 (4)	0.0018 (5)
C32	0.0154 (6)	0.0128 (6)	0.0197 (6)	0.0012 (5)	0.0029 (5)	0.0002 (5)
C33	0.0156 (6)	0.0215 (7)	0.0145 (6)	0.0011 (5)	0.0035 (5)	−0.0012 (5)
C34	0.0170 (6)	0.0210 (7)	0.0158 (6)	−0.0037 (5)	0.0021 (5)	0.0036 (5)
C35	0.0217 (7)	0.0153 (6)	0.0191 (6)	−0.0062 (5)	0.0012 (5)	0.0004 (5)
C36	0.0180 (6)	0.0168 (7)	0.0133 (6)	−0.0028 (5)	0.0018 (5)	−0.0019 (5)
B2	0.0127 (6)	0.0133 (7)	0.0158 (6)	−0.0006 (5)	0.0024 (5)	−0.0026 (5)
C37	0.0156 (6)	0.0146 (6)	0.0197 (6)	−0.0054 (5)	0.0041 (5)	−0.0061 (5)
C38	0.0211 (7)	0.0232 (7)	0.0204 (7)	−0.0070 (6)	0.0041 (5)	−0.0033 (6)
C39	0.0372 (9)	0.0284 (8)	0.0218 (7)	−0.0173 (7)	0.0122 (6)	−0.0069 (6)
C40	0.0298 (8)	0.0272 (8)	0.0463 (10)	−0.0156 (7)	0.0254 (7)	−0.0185 (7)
C41	0.0180 (7)	0.0207 (8)	0.0538 (10)	−0.0053 (6)	0.0144 (7)	−0.0102 (7)
C42	0.0165 (6)	0.0156 (7)	0.0324 (8)	−0.0035 (5)	0.0061 (6)	−0.0049 (6)
C43	0.0099 (6)	0.0146 (6)	0.0193 (6)	−0.0020 (5)	0.0011 (5)	−0.0014 (5)
C44	0.0148 (6)	0.0166 (7)	0.0193 (6)	−0.0011 (5)	0.0033 (5)	−0.0036 (5)
C45	0.0137 (6)	0.0151 (6)	0.0271 (7)	0.0023 (5)	0.0004 (5)	−0.0022 (5)
C46	0.0166 (6)	0.0180 (7)	0.0218 (6)	0.0029 (5)	−0.0036 (5)	0.0024 (5)
C47	0.0157 (6)	0.0225 (7)	0.0164 (6)	0.0011 (5)	−0.0012 (5)	−0.0011 (5)
C48	0.0125 (6)	0.0164 (6)	0.0183 (6)	0.0002 (5)	0.0000 (5)	−0.0035 (5)
C49	0.0164 (6)	0.0130 (6)	0.0163 (6)	−0.0028 (5)	0.0053 (5)	0.0002 (5)
C50	0.0183 (6)	0.0163 (6)	0.0198 (6)	−0.0027 (5)	0.0024 (5)	−0.0004 (5)
C51	0.0275 (7)	0.0182 (7)	0.0196 (6)	−0.0084 (6)	0.0048 (5)	−0.0030 (5)
C52	0.0335 (8)	0.0132 (6)	0.0244 (7)	−0.0037 (6)	0.0129 (6)	−0.0027 (6)
C53	0.0261 (7)	0.0143 (7)	0.0274 (7)	0.0027 (6)	0.0081 (6)	0.0034 (6)
C54	0.0201 (7)	0.0157 (6)	0.0193 (6)	−0.0004 (5)	0.0042 (5)	0.0026 (5)
C55	0.0158 (6)	0.0112 (6)	0.0173 (6)	−0.0007 (5)	0.0014 (5)	0.0006 (5)
C56	0.0174 (6)	0.0142 (6)	0.0181 (6)	−0.0008 (5)	0.0024 (5)	−0.0003 (5)
C57	0.0142 (6)	0.0145 (6)	0.0258 (7)	0.0001 (5)	0.0009 (5)	0.0023 (5)
C58	0.0212 (7)	0.0130 (6)	0.0210 (6)	−0.0028 (5)	−0.0057 (5)	0.0033 (5)
C59	0.0267 (7)	0.0171 (7)	0.0171 (6)	−0.0036 (6)	0.0015 (5)	−0.0026 (5)
C60	0.0187 (6)	0.0168 (7)	0.0205 (6)	−0.0019 (5)	0.0033 (5)	−0.0015 (5)

### Geometric parameters (Å, º)

**Table:**

N1—C1	1.3390 (16)	C25—C30	1.4029 (18)
N1—C2	1.4655 (17)	C25—C26	1.4029 (18)
N1—C3	1.4671 (17)	C26—C27	1.3942 (19)
N2—C1	1.3388 (17)	C26—H26A	0.9500
N2—C5	1.4638 (17)	C27—C28	1.387 (2)
N2—C4	1.4648 (17)	C27—H27A	0.9500
N3—C1	1.3540 (17)	C28—C29	1.390 (2)
N3—C6	1.4630 (16)	C28—H28A	0.9500
N3—C7	1.4697 (17)	C29—C30	1.3928 (19)
N4—C10	1.4983 (17)	C29—H29A	0.9500
N4—C12	1.5006 (16)	C30—H30A	0.9500
N4—C11	1.5027 (16)	C31—C32	1.4039 (18)
N4—C9	1.5171 (16)	C31—C36	1.4051 (18)
C2—H2A	0.9800	C32—C33	1.3941 (19)
C2—H2B	0.9800	C32—H32A	0.9500
C2—H2C	0.9800	C33—C34	1.384 (2)
C3—H3A	0.9800	C33—H33A	0.9500
C3—H3B	0.9800	C34—C35	1.3875 (19)
C3—H3C	0.9800	C34—H34A	0.9500
C4—H4A	0.9800	C35—C36	1.3937 (19)
C4—H4B	0.9800	C35—H35A	0.9500
C4—H4C	0.9800	C36—H36A	0.9500
C5—H5A	0.9800	B2—C43	1.642 (2)
C5—H5B	0.9800	B2—C49	1.6438 (19)
C5—H5C	0.9800	B2—C55	1.6470 (19)
C6—H6A	0.9800	B2—C37	1.6471 (19)
C6—H6B	0.9800	C37—C42	1.4015 (19)
C6—H6C	0.9800	C37—C38	1.403 (2)
C7—C8	1.5389 (17)	C38—C39	1.390 (2)
C7—H7A	0.9900	C38—H38A	0.9500
C7—H7B	0.9900	C39—C40	1.388 (3)
C8—C9	1.5254 (18)	C39—H39A	0.9500
C8—H8A	0.9900	C40—C41	1.380 (3)
C8—H8B	0.9900	C40—H40A	0.9500
C9—H9A	0.9900	C41—C42	1.397 (2)
C9—H9B	0.9900	C41—H41A	0.9500
C10—H10A	0.9800	C42—H42A	0.9500
C10—H10B	0.9800	C43—C48	1.4017 (18)
C10—H10C	0.9800	C43—C44	1.4067 (18)
C11—H11A	0.9800	C44—C45	1.3911 (19)
C11—H11B	0.9800	C44—H44A	0.9500
C11—H11C	0.9800	C45—C46	1.388 (2)
C12—H12A	0.9800	C45—H45A	0.9500
C12—H12B	0.9800	C46—C47	1.387 (2)
C12—H12C	0.9800	C46—H46A	0.9500
B1—C25	1.6427 (19)	C47—C48	1.3963 (19)
B1—C19	1.6451 (19)	C47—H47A	0.9500
B1—C31	1.6584 (19)	C48—H48A	0.9500
B1—C13	1.6600 (19)	C49—C54	1.4051 (19)
C13—C18	1.4034 (18)	C49—C50	1.4088 (18)
C13—C14	1.4116 (19)	C50—C51	1.393 (2)
C14—C15	1.391 (2)	C50—H50A	0.9500
C14—H14A	0.9500	C51—C52	1.390 (2)
C15—C16	1.390 (2)	C51—H51A	0.9500
C15—H15A	0.9500	C52—C53	1.387 (2)
C16—C17	1.382 (2)	C52—H52A	0.9500
C16—H16A	0.9500	C53—C54	1.397 (2)
C17—C18	1.3955 (19)	C53—H53A	0.9500
C17—H17A	0.9500	C54—H54A	0.9500
C18—H18A	0.9500	C55—C60	1.4034 (18)
C19—C24	1.4060 (17)	C55—C56	1.4063 (18)
C19—C20	1.4089 (18)	C56—C57	1.3935 (18)
C20—C21	1.3895 (19)	C56—H56A	0.9500
C20—H20A	0.9500	C57—C58	1.387 (2)
C21—C22	1.3904 (19)	C57—H57A	0.9500
C21—H21A	0.9500	C58—C59	1.386 (2)
C22—C23	1.382 (2)	C58—H58A	0.9500
C22—H22A	0.9500	C59—C60	1.3933 (19)
C23—C24	1.3971 (19)	C59—H59A	0.9500
C23—H23A	0.9500	C60—H60A	0.9500
C24—H24A	0.9500		
			
C1—N1—C2	122.05 (11)	C22—C23—C24	120.35 (12)
C1—N1—C3	122.97 (11)	C22—C23—H23A	119.8
C2—N1—C3	114.96 (11)	C24—C23—H23A	119.8
C1—N2—C5	121.54 (11)	C23—C24—C19	122.82 (12)
C1—N2—C4	123.64 (11)	C23—C24—H24A	118.6
C5—N2—C4	114.80 (11)	C19—C24—H24A	118.6
C1—N3—C6	120.28 (11)	C30—C25—C26	115.54 (12)
C1—N3—C7	122.04 (11)	C30—C25—B1	121.04 (11)
C6—N3—C7	117.52 (11)	C26—C25—B1	123.01 (12)
C10—N4—C12	110.16 (10)	C27—C26—C25	122.68 (13)
C10—N4—C11	108.57 (10)	C27—C26—H26A	118.7
C12—N4—C11	107.94 (10)	C25—C26—H26A	118.7
C10—N4—C9	110.79 (10)	C28—C27—C26	119.96 (13)
C12—N4—C9	110.83 (10)	C28—C27—H27A	120.0
C11—N4—C9	108.45 (10)	C26—C27—H27A	120.0
N2—C1—N1	121.12 (12)	C27—C28—C29	119.18 (13)
N2—C1—N3	118.60 (11)	C27—C28—H28A	120.4
N1—C1—N3	120.28 (12)	C29—C28—H28A	120.4
N1—C2—H2A	109.5	C28—C29—C30	119.99 (13)
N1—C2—H2B	109.5	C28—C29—H29A	120.0
H2A—C2—H2B	109.5	C30—C29—H29A	120.0
N1—C2—H2C	109.5	C29—C30—C25	122.63 (13)
H2A—C2—H2C	109.5	C29—C30—H30A	118.7
H2B—C2—H2C	109.5	C25—C30—H30A	118.7
N1—C3—H3A	109.5	C32—C31—C36	114.81 (12)
N1—C3—H3B	109.5	C32—C31—B1	121.74 (11)
H3A—C3—H3B	109.5	C36—C31—B1	123.41 (11)
N1—C3—H3C	109.5	C33—C32—C31	122.91 (13)
H3A—C3—H3C	109.5	C33—C32—H32A	118.5
H3B—C3—H3C	109.5	C31—C32—H32A	118.5
N2—C4—H4A	109.5	C34—C33—C32	120.45 (12)
N2—C4—H4B	109.5	C34—C33—H33A	119.8
H4A—C4—H4B	109.5	C32—C33—H33A	119.8
N2—C4—H4C	109.5	C33—C34—C35	118.55 (12)
H4A—C4—H4C	109.5	C33—C34—H34A	120.7
H4B—C4—H4C	109.5	C35—C34—H34A	120.7
N2—C5—H5A	109.5	C34—C35—C36	120.34 (13)
N2—C5—H5B	109.5	C34—C35—H35A	119.8
H5A—C5—H5B	109.5	C36—C35—H35A	119.8
N2—C5—H5C	109.5	C35—C36—C31	122.92 (12)
H5A—C5—H5C	109.5	C35—C36—H36A	118.5
H5B—C5—H5C	109.5	C31—C36—H36A	118.5
N3—C6—H6A	109.5	C43—B2—C49	112.26 (10)
N3—C6—H6B	109.5	C43—B2—C55	105.27 (10)
H6A—C6—H6B	109.5	C49—B2—C55	112.44 (10)
N3—C6—H6C	109.5	C43—B2—C37	110.33 (11)
H6A—C6—H6C	109.5	C49—B2—C37	106.20 (10)
H6B—C6—H6C	109.5	C55—B2—C37	110.42 (10)
N3—C7—C8	112.02 (10)	C42—C37—C38	115.43 (13)
N3—C7—H7A	109.2	C42—C37—B2	124.38 (12)
C8—C7—H7A	109.2	C38—C37—B2	120.19 (12)
N3—C7—H7B	109.2	C39—C38—C37	122.67 (14)
C8—C7—H7B	109.2	C39—C38—H38A	118.7
H7A—C7—H7B	107.9	C37—C38—H38A	118.7
C9—C8—C7	108.24 (10)	C40—C39—C38	120.02 (15)
C9—C8—H8A	110.1	C40—C39—H39A	120.0
C7—C8—H8A	110.1	C38—C39—H39A	120.0
C9—C8—H8B	110.1	C41—C40—C39	119.05 (14)
C7—C8—H8B	110.1	C41—C40—H40A	120.5
H8A—C8—H8B	108.4	C39—C40—H40A	120.5
N4—C9—C8	115.25 (10)	C40—C41—C42	120.28 (15)
N4—C9—H9A	108.5	C40—C41—H41A	119.9
C8—C9—H9A	108.5	C42—C41—H41A	119.9
N4—C9—H9B	108.5	C41—C42—C37	122.35 (15)
C8—C9—H9B	108.5	C41—C42—H42A	118.8
H9A—C9—H9B	107.5	C37—C42—H42A	118.8
N4—C10—H10A	109.5	C48—C43—C44	115.20 (12)
N4—C10—H10B	109.5	C48—C43—B2	125.02 (12)
H10A—C10—H10B	109.5	C44—C43—B2	119.76 (11)
N4—C10—H10C	109.5	C45—C44—C43	123.25 (12)
H10A—C10—H10C	109.5	C45—C44—H44A	118.4
H10B—C10—H10C	109.5	C43—C44—H44A	118.4
N4—C11—H11A	109.5	C46—C45—C44	119.58 (13)
N4—C11—H11B	109.5	C46—C45—H45A	120.2
H11A—C11—H11B	109.5	C44—C45—H45A	120.2
N4—C11—H11C	109.5	C47—C46—C45	119.10 (13)
H11A—C11—H11C	109.5	C47—C46—H46A	120.5
H11B—C11—H11C	109.5	C45—C46—H46A	120.5
N4—C12—H12A	109.5	C46—C47—C48	120.43 (13)
N4—C12—H12B	109.5	C46—C47—H47A	119.8
H12A—C12—H12B	109.5	C48—C47—H47A	119.8
N4—C12—H12C	109.5	C47—C48—C43	122.32 (13)
H12A—C12—H12C	109.5	C47—C48—H48A	118.8
H12B—C12—H12C	109.5	C43—C48—H48A	118.8
C25—B1—C19	108.02 (10)	C54—C49—C50	115.02 (12)
C25—B1—C31	108.60 (10)	C54—C49—B2	125.40 (11)
C19—B1—C31	109.35 (10)	C50—C49—B2	119.43 (12)
C25—B1—C13	110.74 (10)	C51—C50—C49	122.77 (13)
C19—B1—C13	111.30 (10)	C51—C50—H50A	118.6
C31—B1—C13	108.78 (10)	C49—C50—H50A	118.6
C18—C13—C14	114.15 (12)	C52—C51—C50	120.28 (13)
C18—C13—B1	123.89 (12)	C52—C51—H51A	119.9
C14—C13—B1	121.89 (11)	C50—C51—H51A	119.9
C15—C14—C13	123.02 (13)	C53—C52—C51	118.84 (13)
C15—C14—H14A	118.5	C53—C52—H52A	120.6
C13—C14—H14A	118.5	C51—C52—H52A	120.6
C16—C15—C14	120.59 (13)	C52—C53—C54	120.16 (13)
C16—C15—H15A	119.7	C52—C53—H53A	119.9
C14—C15—H15A	119.7	C54—C53—H53A	119.9
C17—C16—C15	118.31 (13)	C53—C54—C49	122.90 (13)
C17—C16—H16A	120.8	C53—C54—H54A	118.6
C15—C16—H16A	120.8	C49—C54—H54A	118.6
C16—C17—C18	120.38 (13)	C60—C55—C56	115.11 (12)
C16—C17—H17A	119.8	C60—C55—B2	123.72 (12)
C18—C17—H17A	119.8	C56—C55—B2	121.08 (11)
C17—C18—C13	123.41 (13)	C57—C56—C55	122.83 (13)
C17—C18—H18A	118.3	C57—C56—H56A	118.6
C13—C18—H18A	118.3	C55—C56—H56A	118.6
C24—C19—C20	114.76 (12)	C58—C57—C56	120.06 (13)
C24—C19—B1	123.37 (11)	C58—C57—H57A	120.0
C20—C19—B1	121.83 (11)	C56—C57—H57A	120.0
C21—C20—C19	123.05 (12)	C59—C58—C57	118.84 (12)
C21—C20—H20A	118.5	C59—C58—H58A	120.6
C19—C20—H20A	118.5	C57—C58—H58A	120.6
C20—C21—C22	120.20 (13)	C58—C59—C60	120.40 (13)
C20—C21—H21A	119.9	C58—C59—H59A	119.8
C22—C21—H21A	119.9	C60—C59—H59A	119.8
C23—C22—C21	118.81 (12)	C59—C60—C55	122.62 (13)
C23—C22—H22A	120.6	C59—C60—H60A	118.7
C21—C22—H22A	120.6	C55—C60—H60A	118.7
			
C5—N2—C1—N1	−150.00 (13)	C13—B1—C31—C36	131.83 (12)
C4—N2—C1—N1	31.57 (19)	C36—C31—C32—C33	−1.25 (18)
C5—N2—C1—N3	29.97 (18)	B1—C31—C32—C33	−179.20 (12)
C4—N2—C1—N3	−148.46 (12)	C31—C32—C33—C34	0.1 (2)
C2—N1—C1—N2	−150.60 (12)	C32—C33—C34—C35	1.2 (2)
C3—N1—C1—N2	27.44 (19)	C33—C34—C35—C36	−1.3 (2)
C2—N1—C1—N3	29.43 (18)	C34—C35—C36—C31	0.1 (2)
C3—N1—C1—N3	−152.53 (12)	C32—C31—C36—C35	1.12 (19)
C6—N3—C1—N2	44.91 (17)	B1—C31—C36—C35	179.04 (12)
C7—N3—C1—N2	−139.78 (12)	C43—B2—C37—C42	12.69 (17)
C6—N3—C1—N1	−135.12 (13)	C49—B2—C37—C42	−109.20 (14)
C7—N3—C1—N1	40.19 (18)	C55—B2—C37—C42	128.64 (13)
C1—N3—C7—C8	122.96 (13)	C43—B2—C37—C38	−167.47 (12)
C6—N3—C7—C8	−61.61 (15)	C49—B2—C37—C38	70.64 (15)
N3—C7—C8—C9	−160.81 (11)	C55—B2—C37—C38	−51.52 (16)
C10—N4—C9—C8	71.23 (14)	C42—C37—C38—C39	−4.5 (2)
C12—N4—C9—C8	−51.38 (14)	B2—C37—C38—C39	175.68 (13)
C11—N4—C9—C8	−169.70 (11)	C37—C38—C39—C40	1.2 (2)
C7—C8—C9—N4	−170.40 (10)	C38—C39—C40—C41	2.5 (2)
C25—B1—C13—C18	137.19 (12)	C39—C40—C41—C42	−2.7 (2)
C19—B1—C13—C18	17.01 (17)	C40—C41—C42—C37	−0.8 (2)
C31—B1—C13—C18	−103.53 (14)	C38—C37—C42—C41	4.2 (2)
C25—B1—C13—C14	−46.18 (16)	B2—C37—C42—C41	−175.91 (13)
C19—B1—C13—C14	−166.35 (11)	C49—B2—C43—C48	−14.96 (17)
C31—B1—C13—C14	73.10 (15)	C55—B2—C43—C48	107.68 (13)
C18—C13—C14—C15	4.09 (19)	C37—B2—C43—C48	−133.20 (12)
B1—C13—C14—C15	−172.85 (12)	C49—B2—C43—C44	166.63 (11)
C13—C14—C15—C16	−1.8 (2)	C55—B2—C43—C44	−70.74 (14)
C14—C15—C16—C17	−1.4 (2)	C37—B2—C43—C44	48.39 (15)
C15—C16—C17—C18	2.0 (2)	C48—C43—C44—C45	3.72 (19)
C16—C17—C18—C13	0.5 (2)	B2—C43—C44—C45	−177.71 (12)
C14—C13—C18—C17	−3.47 (19)	C43—C44—C45—C46	−3.1 (2)
B1—C13—C18—C17	173.40 (12)	C44—C45—C46—C47	−0.1 (2)
C25—B1—C19—C24	−3.37 (16)	C45—C46—C47—C48	2.4 (2)
C31—B1—C19—C24	−121.39 (13)	C46—C47—C48—C43	−1.7 (2)
C13—B1—C19—C24	118.40 (13)	C44—C43—C48—C47	−1.27 (18)
C25—B1—C19—C20	174.10 (11)	B2—C43—C48—C47	−179.75 (12)
C31—B1—C19—C20	56.08 (15)	C43—B2—C49—C54	129.63 (13)
C13—B1—C19—C20	−64.13 (15)	C55—B2—C49—C54	11.14 (18)
C24—C19—C20—C21	0.12 (19)	C37—B2—C49—C54	−109.72 (14)
B1—C19—C20—C21	−177.55 (12)	C43—B2—C49—C50	−55.04 (15)
C19—C20—C21—C22	−1.0 (2)	C55—B2—C49—C50	−173.52 (11)
C20—C21—C22—C23	1.0 (2)	C37—B2—C49—C50	65.62 (15)
C21—C22—C23—C24	−0.2 (2)	C54—C49—C50—C51	−1.45 (19)
C22—C23—C24—C19	−0.8 (2)	B2—C49—C50—C51	−177.25 (12)
C20—C19—C24—C23	0.77 (19)	C49—C50—C51—C52	0.2 (2)
B1—C19—C24—C23	178.40 (12)	C50—C51—C52—C53	1.4 (2)
C19—B1—C25—C30	−79.63 (14)	C51—C52—C53—C54	−1.6 (2)
C31—B1—C25—C30	38.87 (16)	C52—C53—C54—C49	0.3 (2)
C13—B1—C25—C30	158.26 (11)	C50—C49—C54—C53	1.20 (19)
C19—B1—C25—C26	92.75 (14)	B2—C49—C54—C53	176.72 (13)
C31—B1—C25—C26	−148.75 (12)	C43—B2—C55—C60	100.23 (14)
C13—B1—C25—C26	−29.36 (17)	C49—B2—C55—C60	−137.25 (13)
C30—C25—C26—C27	−0.1 (2)	C37—B2—C55—C60	−18.83 (17)
B1—C25—C26—C27	−172.88 (13)	C43—B2—C55—C56	−76.22 (15)
C25—C26—C27—C28	0.8 (2)	C49—B2—C55—C56	46.29 (16)
C26—C27—C28—C29	−0.6 (2)	C37—B2—C55—C56	164.71 (12)
C27—C28—C29—C30	−0.1 (2)	C60—C55—C56—C57	4.0 (2)
C28—C29—C30—C25	0.8 (2)	B2—C55—C56—C57	−179.26 (12)
C26—C25—C30—C29	−0.66 (19)	C55—C56—C57—C58	−2.1 (2)
B1—C25—C30—C29	172.25 (12)	C56—C57—C58—C59	−1.4 (2)
C25—B1—C31—C32	70.22 (14)	C57—C58—C59—C60	2.6 (2)
C19—B1—C31—C32	−172.13 (11)	C58—C59—C60—C55	−0.4 (2)
C13—B1—C31—C32	−50.39 (15)	C56—C55—C60—C59	−2.8 (2)
C25—B1—C31—C36	−107.56 (13)	B2—C55—C60—C59	−179.40 (13)
C19—B1—C31—C36	10.09 (16)		

### Hydrogen-bond geometry (Å, º)

Cg1, Cg4, Cg5, Cg6, and Cg8 are the centroids of the C13–C18,C31–C36, C37–C42, C43–C48 and C55–C60 rings, respectively.

**Table:**

*D*—H···*A*	*D*—H	H···*A*	*D*···*A*	*D*—H···*A*
C3—H3*C*···*Cg*7	0.98	2.62	3.3783 (16)	134
C7—H7*B*···*Cg*1	0.99	2.80	3.7805 (14)	169
C9—H9*B*···*Cg*6	0.99	2.52	3.4075 (14)	149
C11—H11*C*···*Cg*5^i^	0.98	2.62	3.4852 (15)	147
C12—H12*A*···*Cg*4	0.98	2.59	3.4044 (15)	141
C12—H12*B*···*Cg*8^ii^	0.98	2.69	3.5990 (15)	155

Symmetry codes: (i) −*x*+1, *y*+1/2, −*z*+1/2; (ii) *x*, −*y*+3/2, *z*+1/2.
